# The associated factors of cesarean section during COVID-19 pandemic: a cross-sectional study in nine cities of China

**DOI:** 10.1186/s12199-020-00899-w

**Published:** 2020-10-10

**Authors:** Jian Zhang, Yumei Zhang, Yidi Ma, Yalei Ke, Shanshan Huo, Liping He, Wenjuan Luo, Jing Li, Ai Zhao

**Affiliations:** 1 Vanke School of Public Health, Tsinghua University, Beijing, 100091 China; 2 School of Public Health, Peking University, Beijing, 100191 China; 3 School of Public Health, XiangNan University, Chenzhou, 423000 China; 4 Jiangxi Maternal and Child Health Hospital, Nanchang, 330006 China; 5 Shenyang Maternal and Child Health Hospital, Shenyang, 110000 China

**Keywords:** COVID-19, Pregnancy, Cesarean section, Gestational weight gain

## Abstract

**Background:**

Improving and maintaining the health of mothers and newborns is indisputably a global priority, especially during a pandemic. This study intends to examine the factors associated with cesarean section (CS) during lockdown time.

**Methods:**

A total of 678 women who just gave birth within 7 days were enrolled from maternal and children hospitals in nine cities of China from April to May 2020. The delivery modes and potential influencing factors were investigated. The subgroup analysis and sensitivity analysis were used to examine the association of CS and risk factors among populations with different characteristics and to control for possible confounding, respectively.

**Results:**

The overall rate of cesarean delivery was 37.3%. In multi-variant model, maternal age > 30 years (OR, 95% CI = 1.71, 1.21–2.41), higher pre-gestational BMI (OR, 95% CI = 1.16, 1.10–1.23), living in regions with confirmed COVID-19 cases > 500 (OR, 95% CI = 2.45, 1.74–3.45), and excess gestational weight gain (OR, 95% CI = 1.73, 1.17–2.55) were associated with cesarean delivery. These trends of associations were not changes in sensitivity analysis and subgroup analysis. Cesarean delivery occurred more in women who got more nutrition instruction during the pandemic period in the univariant model; however, this association showed insignificance in the multiple-variant analysis.

**Conclusion:**

A high cesarean delivery rate was found in uninfected women who experienced lockdown in their third trimester. During the COVID-19 pandemic, more medical support should be provided in severely affected regions to ensure and promote health in pregnancy.

## Introduction

The 2019 novel coronavirus (COVID-19), a severe acute respiratory syndrome caused by a coronavirus, was declared as a pandemic by the World Health Organization (WHO) on March 11, 2020 [1], due to its highly contagious nature, subsequently spreading around the world and infecting more than 13 million confirmed cases by July 15 [2]. This is still true today as the world continues to grapple with the COVID-19 pandemic. China and many countries have adopted several measures to control disease transmission, including travel restrictions, early detection, isolation of suspected and confirmed cases, and widespread quarantines [3, 4].

Improving the survival and well-being of mothers and newborns is indisputably a global priority, especially during pandemic [5]. Since COVID-19 broke out, there has been plenty of research focused on pregnant women infected with COVID-19 [6–8]. For maternal and newborn health, however, a critical question today is not only the extent to which pregnant or postpartum women and newborns are vulnerable to COVID-19 infection but also the degree to which giving a healthy birth in the general pregnant population who are not infected with coronavirus is influenced by lockdown and quarantines. The impact of COVID-19 on pregnancy outcomes among uninfected women has not been acknowledged up to this point. Studies have shown that large scale emergencies have increased morbidity and often mortality in women and infants [9–11]. In addition, during this time, pregnant women need prenatal care more than ever in order to monitor their health and well-being, answer their questions, check on the progress of their pregnancy, and avoid any adverse pregnancy outcomes [12]. However, regular prenatal examinations have been more or less impacted by the pandemic [13], which has put pregnant women at a higher risk of poor perinatal health management and may lead to series of adverse pregnancy outcomes.

There are around 15 million births in 2018 in China [14]. The COVID-19 pandemic has caused numerous challenges in ensuring health in pregnancy. In this study, delivery modes and other pregnancy outcomes were collected from new mothers who experienced lockdown during their third trimester in China (January to April 2020) and to explore its associated factors.

## Methods

### Participants

This cross-sectional study was conducted in China during the COVID-19 outbreak between April and May 2020. A multi-sampling strategy was used to recruit new mothers who had just given birth within 7 days. Firstly, we purposely selected nine cities in China according to their geographic location and economic status (Wuhan, Beijing, Xiangyang, Nanchang, Suzhou, Xuchang, Tangshan, Cangzhou, and Lanzhou). Secondly, one hospital in each city was selected for convenience. Then, women who met the inclusion criteria were invited by local health professionals and voluntarily participated in this study. The inclusion criteria were as follows: (1) gave birth within the past 7 days, (2) single birth, (3) lived in the investigated regions during the third trimester, and (4) were aged from 18 to 45 years. The women who confirmed infection with COVID-19 or any other severe disease (e.g., cancer, severe infection, or liver or kidney failure) and the ones who gave birth to a baby with a birth defect were excluded from the study. Finally, the data of 678 participants was used in the analysis.

### Data collection

The data were collected with one e-questionnaire using a cellphone-based tool (Wenjuan xing Tech Co. Ltd, Changsha, China). The questionnaire included four parts: sociodemographic characteristics, information on delivery modes and pregnancy and neonatal health, physical activities, and the medical services which women accessed during the lockdown period.

Sociodemographic characteristics of participants included age, average monthly family income, and education level. The cumulative numbers of confirmed COVID-19 cases (by May 25) in the provinces where our studied cities were located was obtained from the Distribution of COVID-19 Report [15]. Participants were also required to score their degree of concern on COVID-19 (from zero to ten, where zero indicated not worried at all, and 10 indicated severely worried about COVID-19).

Delivery modes were investigated and the pregnancy information includes parity, gestational weeks, self-reported maternal weight measured before pregnancy, and at the last prenatal examination before delivery, maternal complications and disease occurred during pregnancy (including gestational diabetes, dyslipidemia, and hypothyroidism) were obtained. Women with any maternal complications and diseases mentioned above were considered as “having metabolic disease”. Women who could not clearly recall the information were suggested to ask their doctors in charge. The frequency of taking physical activity every week was also obtained.

Pre-gestational body mass index (BMI) was calculated as pre-gestational weight (kg) divided by square of height (m^2^). The gestational weight gain (GWG) was defined as the difference between weight before labor and pre-gestational weight. Optimal weight gain was defined by the 2009 Institute of Medicine (IOM) guidelines, and women who had GWG lower or higher than the recommended values were classified as low or excess GWG, respectively [16]. Gestational weeks < 37 weeks were considered as preterm birth, while gestational age ≥ 42 weeks was considered as post-term birth. The definition of macrosomia was a neonatal birth weight > 4000 g, and low birth weight (LBW) was defined as < 2500 g [17, 18].

With regard to medical services, whether women regularly attended the prenatal check-ups during lockdown time was investigated and categorized as “never,” “not attend regular as doctor suggested,” and “regular attend”. The ways women got nutrition instruction was also investigated with a multiple-choice question (nutrition clinic of the hospital, obstetrics clinic, online instruction, and online courses); individuals who got any instructions through the four approaches were regarded as getting nutrition instruction during lockdown period.

### Statistics

Results were presented as means and SDs for continuous variables and percentages for categorical variables. Differences across groups were compared by Student’s *t* tests and Chi-squared tests for continuous and categorical variables, respectively. Multivariate logistic regression models were carried out to investigate influence factors on the delivery mode of participants. Factors with *P* < 0.05 in the univariate analysis were included in the multivariate model, including age (≤ 30 or > 30 years), pre-gestational BMI (continuous), number of COVID-19 cases in the resident region (≤ 500 or > 500), history of metabolic disease (no or yes), getting nutrition instruction during lockdown period (no or yes), and GWG (optimal, low, or excess). Sensitivity analysis was conducted by additionally including education level (middle school and below, or college and above) and getting regular prenatal check-ups during the lockdown period (no or yes) in the multivariate model. Subgroup analyses were conducted by excluding participants whose infants were preterm or post-term, whose infants had macrosomia and whose infant’s birth weight was missing, or who had metabolic diseases in pregnancy. All the statistics were conducted in R 4.0.2. All *P* values were two-sided. Statistical significance was defined as *P* < 0.05.

## Results

### General characteristics of participants

In the study population, 37.3% and 62.7% gave cesarean delivery and vaginal delivery, respectively. Table 1 presents participants’ general characteristics according to delivery modes. Participants who had a cesarean section (CS) were more likely to be over 30 years old, having higher pre-gestational BMI, and be living in regions with over 500 COVID-19 cases. Participants with different education levels, income, exercise frequency, and degree of concerns on COVID-19 did not differ significantly in delivery modes.

**Table 1:** General characteristics between participants with different delivery modes

	Vaginal	Cesarean	*P*
Age (years)			
≤ 30	290 (68.2)	135 (53.4)	< 0.001
> 30	135 (31.8)	118 (46.6)	
Education level			
Middle school and below	110 (25.9)	84 (33.2)	0.051
College and above	315 (74.1)	169 (66.8)	
Income^a^			
< 5000	107 (27.2)	80 (34.2)	0.150
5000 to < 10,000	132 (33.6)	76 (32.5)	
≥ 10000	154 (39.2)	78 (33.3)	
Exercise frequency (times per week)			
≤ 1	74 (17.4)	44 (17.4)	0.931
2–3	114 (26.8)	69 (27.3)	
4–6	56 (13.2)	29 (11.5)	
7	181 (42.6)	111 (43.9)	
Pre-gestational BMI (kg/m^2^)	20.8 ± 2.8	22.3 ± 3.5	< 0.001
Number of COVID-19 cases in the resident region			
≤ 500	249 (58.6)	92 (36.4)	< 0.001
> 500	176 (41.4)	161 (63.6)	
Degree of concerns on COVID-19	6.1 ± 2.6	6.0 ± 2.8	0.739

Continuous variables were presented as mean ± SD and compared with Student’s *t* tests. Categorical variables were presented as proportions and compared with Chi-squared tests

### Information on health and delivery

Participants with CS were more likely to get nutrition instruction during the pandemic, have a history of metabolic disease, and have excess GWG. No association was observed between delivery modes with parities, getting regular pregnancy check-ups, gestational age at delivery, or birth weight of infants (Table 2).

**Table 2:** Differences of health and pregnancy information between participants with different delivery modes

	Vaginal	Cesarean	*P*
Parities			
First birth	248 (58.4)	139 (54.9)	0.431
Others	177 (41.6)	114 (45.1)	
Getting regular pregnancy check-ups			
No	213 (50.1)	109 (43.1)	0.090
Yes	212 (49.9)	144 (56.9)	
Getting nutrition instruction			
No	161 (37.9)	73 (28.9)	0.021
Yes	264 (62.1)	180 (71.1)	
History of metabolic disease			
No	356 (83.8)	187 (73.9)	0.003
Yes	69 (16.2)	66 (26.1)	
Gestational weight gain			
Low	101 (23.8)	59 (23.3)	0.013
Optimal	200 (47.1)	94 (37.2)	
Excess	124 (29.2)	100 (39.5)	
Gestational age at delivery			
Full-term	402 (94.6)	235 (92.9)	0.618
Pre-term	21 (4.9)	17 (6.7)	
Post-term	2 (0.5)	1 (0.4)	
Birth weight of infants (kg)^a^	3.3 ± 0.4	3.3 ± 0.6	0.832

Continuous variables were presented as mean ± SD and compared with Student’s *t* tests. Categorical variables were presented as proportions and compared with Chi-square tests

^a^One missing value

Considering the different ways women accessed to nutrition instruction, the top three ways were obstetrics clinic (44.5%), online instruction (33.6%), and online courses (19.2%), respectively. Participants living in areas with over 500 COVID-19 cases were more likely to get more nutrition instructions from online instruction (e.g., using apps on cellphones) and online courses (Supplementary Table 1).

### Multivariate analysis

Multivariate analysis showed that being over 30 years old, having higher pre-gestational BMI, living in regions with over 500 COVID-19 cases, and have excess GWG were associated with CS. The associations of history of metabolic disease and getting nutrition instructions with delivery mode were not significant (Table 3).

**Table 3:** Multivariate analysis of associated factors of delivery mode

Variables	Crude		Multivariate	
	OR (95% CI)	*P*	OR (95% CI)	*P*
Age (years)				
≤ 30	Ref		Ref	
> 30	1.88 (1.36, 2.59)	< 0.001	1.71 (1.21, 2.41)	0.002
Pre-gestational BMI (kg/m^2^)	1.18 (1.12, 1.25)	< 0.001	1.16 (1.10, 1.23)	< 0.001
Number of COVID-19 cases in the resident region				
≤ 500	Ref		Ref	
> 500	2.48 (1.80, 3.42)	< 0.001	2.45 (1.74, 3.45)	< 0.001
History of metabolic disease				
No	Ref		Ref	
Yes	1.82 (1.24, 2.67)	0.002	1.41 (0.93, 2.13)	0.103
Getting nutrition instruction				
No	Ref		Ref	
Yes	1.50 (1.08, 2.11)	0.017	1.35 (0.94, 1.95)	0.107
Gestational weight gain				
Optimal	Ref		Ref	
Low	1.24 (0.83, 1.86)	0.291	1.26 (0.82, 1.94)	0.285
Excess	1.72 (1.20, 2.46)	0.003	1.73 (1.17, 2.55)	0.006

Factors with *P* < 0.05 in the univariate analysis were included in the multivariate logistic regression model

*BMI* body mass index, *COVID-19* 2019 novel coronavirus, *OR* odds ratio, *Ref* reference

### Sensitivity analysis and subgroup analysis

In the sensitivity analysis, the associations of age, pre-gestational BMI, number of COVID-19 cases in the resident region, and GWG with delivery modes did not change after additionally including education level and getting regular prenatal check-ups in the multivariate model (Supplementary Table 2). The association also did not change in the subgroup analyses by excluding participants whose infants were preterm or post-term, whose infants had macrosomia and whose infant’s birth weight was missing, or who had metabolic diseases in pregnancy (Table 4).

**Table 4:** Subgroup analysis of multivariate analysis on exploring associated factors of delivery mode

Variables	Model 1	Model 2	Model 3
Number of cases	637	643	543
Age (years)			
≤ 30	Ref	Ref	Ref
> 30	1.67 (1.17, 2.38)	1.83 (1.29, 2.61)	1.83 (1.24, 2.70)
Pre-gestational BMI (kg/m^2^)	1.16 (1.09, 1.23)	1.17 (1.10, 1.24)	1.16 (1.09, 1.24)
Number of COVID-19 cases in resident region			
≤ 500	Ref	Ref	Ref
> 500	2.48 (1.75, 3.53)	2.54 (1.79, 3.63)	2.72 (1.85, 4.03)
History of metabolic disease			
No	Ref	Ref	Ref
Yes	1.31 (0.86, 2.01)	1.31 (0.85, 2.01)	-
Getting nutrition instruction			
No	Ref	Ref	Ref
Yes	1.22 (0.84, 1.77)	1.36 (0.93, 2.00)	1.13 (0.75, 1.70)
Gestational weight gain			
Optimal	Ref	Ref	Ref
Low	1.23 (0.79, 1.92)	1.27 (0.81, 1.97)	1.01 (0.61, 1.64)
Excess	1.74 (1.17, 2.59)	1.79 (1.20, 2.68)	1.70 (1.10, 2.64)

Multivariate logistic regression models were conducted to estimate odds ratios

Model 1: Participants with preterm (*N* = 38) or post-term (*N* = 3) birth were excluded

Model 2: Participants with fetal macrosomia (*N* = 34) or whose infant’s birth weight was missing (*N* = 1) were excluded

Model 3: Participants with metabolic diseases were excluded (*N* = 135)

*BMI* body mass index, *COVID-19* 2019 novel coronavirus, *OR* odds ratio, *Ref* reference

## Discussion

In recent decades, psychosocial and sociocultural factors have been increasingly incorporated into theory on pregnancy in order to improve the scientific understanding of the factors that elevate or reduce reproductive risk [19]. With the subsequent development of a global pandemic, COVID-19 has significantly changed the lifestyles of pregnant women, raises anxiety and concerns, and bringing great impacts on their health management [13, 20]. With a multi-center design, this study is the first to report that there was a relatively high CS rate in China during the pandemic, especially in the areas that were severely affected by the disease.

When medically justified, a CS can prevent maternal and perinatal mortality and morbidity. However, currently available evidence shows that there was no important association between the CS rate and maternal and neonatal mortality when the CS rate exceeded 20% [21]. The WHO recommends that there is no justification for any region to have a CS rate higher than 10–15% [22]. Although China is considered as the country with the highest CS rate [23], according to Xinhua News reports, this is attributed to the government’s measures to control unnecessary birth surgeries; in megacities, there is an average 2.1% drop in the CS rate each year, while the percentage in most other cities remains stable [24]. In this study, we found CS rate is 37.3%, which is similar with it reported in pre-COVID-19 era (2018, 36.7%) [25]. However, we should pay attention; this study showed relatively high CS rates in severely affected regions. It is conceivable that the COVID-19 outbreak has more or less led to the current reported high CS rate.

Not surprisingly, more confirmed COVID-19 cases could bring more restrictions on daily life. Our previous study once reported a lower dietary diversity in populations living in severely affected areas than people living in places with less confirmed cases [26]. We also infer that women in severely affected places may be required to “shelter at home” and may have missed more prenatal examinations [5]. The current study also revealed a significantly higher percentage of “never attending” the regular prenatal check-ups by women living in the regions with more confirmed cases (8.9%) than by women living in less confirmed cases regions (2.3%). Further studies needed to explore the underlying reasons led to the skip of regular check-ups. In addition, one study also suggested the lockdown policy may interfere with the circadian rhythm of women and fetuses, which may have led to several adverse pregnancy outcomes and may result in CS [27]. These results advocate that more medical support and more targeted guidance should be implemented in severely affected regions.

Another important contributor to CS is GWG. In this study, we found that women who had excess GWG had 1.7 times higher risk of experiencing CS, and this result did not change its trend in the sensitivity analysis and subgroup analysis, which indicate the results are quite stable. This finding concurs with many previous studies conducted in different populations, including China [28–30]. According to a study conducted during the pandemic, people tend to put on weight caused by lack of enough physical activities and due to eating more food [31]. As a psychologist has warned, emotional eating easily occurs when people are under great social stress, e.g., COVID-19 outbreak, and may lead to failure of weight management [32]. Our previous study also confirmed, during pandemic, that emotional eating contributed to an excessive weight gain [33]. Although physical activities were found not associated with C-section in this study, we should notice that the moderate exercise could help manage an appropriate weight gain which could help to reduce the risk of CS [34]. Besides elevating the risk of CS, excess GWG could also bring lots of adverse pregnancy outcomes, such as macrosomia [35]. Further studies are needed to explore the risk factors that impact on maternal health management during disease outbreak and to provide more specific recommendations.

In the univariate analysis, we observed higher CS rate positively associated with a higher chance of getting nutrition instruction by health professionals and a marginally higher percentage to regularly attend the prenatal check-ups; however, these associations disappeared in the multivariate model, which adjusted for maternal age, pre-gestational BMI, and GWG. These findings may indicate that during lockdown time, prenatal health care services indeed reached women with higher risk. However, we found there were still great proportion of women that did not regularly attend the prenatal check-ups. When the regions are severely affected by disease, and it is hard to access the clinics due to lockdown policies, the online medical services may be one of the options. Mobile health clinics are a model that has been shown to be effectively used in emergency situations when traditional health care is disrupted [36]. In this study, we also observed women living in regions where confirmed cases over 500 were more likely to get nutrition instruction from online services. However, a small proportion of in-person visits were still necessary, such as for monitoring the fetus’s heartbeat and measuring the blood pressure. One US study also attempted to develop a drive-through prenatal care model in response to the coronavirus [37]. Appropriate health care models need to be explored to cope with the unprecedented challenges in accommodating the additional need to minimize the risk of COVID-19 exposure.

## Limitations

There is no doubt of the causality between pregnancy characteristics and pregnancy outcomes in the current survey. However, as with any cross-sectional studies, the limitations of retrospective design are unavoidable, and the results should be generalized with caution. Recall bias might exist, especially on physical activities and pre-gestational weight. Although the multi-variant models and sensitivity analysis strategies were used to control the potential confounding factors such as metabolic disease, the residual confounding by unmeasured factors remains possible for the current findings. In addition, there are complex of factors that impact on pregnancy outcomes, such as nutrition and psychological factors. These factors may also be greatly affected by the lockdown; however, unfortunately, these variables were not measured in the current study. For measuring the severity of COVID-19, it should be noticed that in this study, the number of confirmed cases was from provincial level; the exact confirmed cases in city level cannot be accessed to. And since this study was conducted in urban areas, how COVID-19 affects the rural areas is still unknown and requires further research.

## Conclusion

This study reported the CS rate is 37.3% among uninfected women living in urban areas of China. Besides maternal age and pre-gestational BMI, women living in severely affected regions and having an excess GWG were identified as being associated with a higher risk of CS. This study supports the need for more medical support to be conducted in severely affected areas. A dearth of studies on pregnancy health during the COVID-19 pandemic advocates further studies to confirm health threat on women and neonatal and to provide a more targeted support.

## Supplementary information

**Additional file 1: Supplementary Table 1.** Ways of women access to nutrition instructions among women living in the regions with different COVID-19 confirmed cases. **Supplementary Table 2.** Sensitivity analysis of influence factors of delivery mode.^a^

## Data Availability

The datasets analyzed in this study are available upon request to the corresponding author.

## Abbreviations

COVID-19: 2019 novel coronavirus
WHO: World Health Organization
BMI: Body mass index
GWG: Gestational weight gain
LBW: Low birth weight
CS: Cesarean section

## References

[1] Centers for Disease Control and Prevention: Situation Summary. https://www.cdc.gov/coronavirus/2019-ncov/cases-updates/summary.html?CDC_AA_refVal=https%3A%2F%2Fwww.cdc.gov%2Fcoronavirus%2F2019-ncov%2Fsummary.html#emergence. Accessed 15 Jul 2020.

[2] World Health Organization: Coronavirus disease (COVID-2019) situation reports. https://www.who.int/emergencies/diseases/novel-coronavirus-2019/situation-reports. Accessed 16 Apr 2020.

[3] Zhong BL, Luo W, Li HM, Zhang QQ, Liu XG, Li WT (2020). Knowledge, attitudes, and practices towards COVID-19 among Chinese residents during the rapid rise period of the COVID-19 outbreak: a quick online cross-sectional survey. Int J Biol Sci..

[4] Xiang YT, Yang Y, Li W, Zhang L, Zhang Q, Cheung T (2020). Timely mental health care for the 2019 novel coronavirus outbreak is urgently needed. Lancet Psychiatry..

[5] Graham WJ, Afolabi B, Benova L, Campbell OMR, Filippi V, Nakimuli A (2020). Protecting hard-won gains for mothers and newborns in low-income and middle-income countries in the face of COVID-19: call for a service safety net. BMJ Glob Health..

[6] González Romero D, Ocampo Pérez J, González Bautista L, Santana-Cabrera L. Pregnancy and perinatal outcome of a woman with COVID-19 infection. Rev Clin Esp. 2020. 10.1016/j.rce.2020.04.006. 10.1016/j.rce.2020.04.006 PMC7164884 38620169

[7] Nawsherwan KS, Nabi G, Fan C, Wang S (2020). Impact of COVID-19 pneumonia on neonatal birth outcomes. Indian J Pediatr.

[8] Rozycki HJ, Kotecha S. Covid-19 in pregnant women and babies: what pediatricians need to know. Paediatric Respiratory Reviews. 2020. 10.1016/j.prrv.2020.06.006. 10.1016/j.prrv.2020.06.006 PMC7293440 32709462

[9] Al Gasseer N, Dresden E, Keeney GB, Warren N (2004). Status of women and infants in complex humanitarian emergencies. J Midwifery Womens Health..

[10] Brennan RJ, Nandy R (2001). Complex humanitarian emergencies: a major global health challenge. Emerg Med (Fremantle)..

[11] Keely C, Reed H, Waldman R (2001). Understanding mortality patterns in complex humanitarian emergencies. Forced Migration & Mortality.

[12] Fryer K, Delgado A, Foti T, Reid CN, Marshall J. Implementation of obstetric telehealth during COVID-19 and beyond. Matern Child Health J. 2020:1–7. 10.1007/s10995-020-02967-7. 10.1007/s10995-020-02967-7 PMC7305486 32564248

[13] Tang K, Gaoshan J, Ahonsi B, Ali M, Bonet M, Broutet N (2020). Sexual and reproductive health (SRH): a key issue in the emergency response to the coronavirus disease (COVID-19) outbreak. Reprod Health..

[14] National Bureau of Statistic. China Statistical Yearbook. Available from: http://www.stats.gov.cn/tjsj/ndsj/ . Accessed 28 May 2020.

[15] National Health Commission of People’s Republic of China: Coronavirus disease situation reports. http://www.nhc.gov.cn/xcs/yqtb/list_gzbd.shtml. Accessed 28 May 2020.

[16] Institute of Medicine (US) and National Research Council (US) Committee to Reexamine IOM Pregnancy Weight Guidelines (2009). Weight gain during pregnancy: reexamining the guidelines.

[17] Sun Y, Shen Z, Zhan Y, Wang Y, Ma S, Zhang S (2020). Effects of pre-pregnancy body mass index and gestational weight gain on maternal and infant complications. BMC Pregnancy Childbirth..

[18] Galal M, Symonds I, Murray H (2012). Postterm pregnancy. Facts Views & Vision in Obgyn.

[19] Baum A, Revenson TA, Singer JE (2012). Handbook of health psychology.

[20] Rashidi Fakari F, Simbar M (2020). Coronavirus pandemic and worries during pregnancy; a letter to Editor. Arch Acad Emerg Med..

[21] Molina G, Weiser TG , Lipsitz SR , etal. Relationship between cesarean delivery rate and maternal and neonatal mortality. J Am Med Assoc, 2015, 314:2263-2270. doi: 10.1001/jama.2015.15553. 10.1001/jama.2015.15553 26624825

[22] World Health Organization (1985). Appropriate technology for birth. Lancet..

[23] Mi J, Liu F (2014). Rate of caesarean section is alarming in China. Lancet..

[24] Li Y: Cesarean section rate rises in China: report. http://www.ecns.cn/news/2020-01-10/detail-ifzsqcrm6564120.shtml. .

[25] Department of Maternal and Child Health, National Health Commission. China maternal and child health development report (2019). http://www.nhc.gov.cn/fys/s7901/201905/bbd8e2134a7e47958c5c9ef032e1dfa2.shtml. Accessed 31 Aug 2020.

[26] Zhao A, Li Z, Ke Y, Huo S, Ma Y, Zhang Y (2020). Dietary diversity among Chinese residents during the COVID-19 outbreak and its associated factors. Nutrients..

[27] Bagci S, Sabir H, Müller A, Reiter RJ. Effects of altered photoperiod due to COVID-19 lockdown on pregnant women and their fetuses. Chronobiol Int. 2020. 10.1080/07420528.2020.1772809.1-13. 10.1080/07420528.2020.1772809 32519912

[28] Hawley NL, Johnson W, Hart CN, Triche EW, Ah Ching J, Muasau-Howard B (2015). Gestational weight gain among American Samoan women and its impact on delivery and infant outcomes. BMC Pregnancy Childbirth..

[29] Godoy AC, Nascimento SL, Kasawara KT, Hatsue Oushiro N, Surita FG. A population-based study on gestational weight gain according to body mass index in the Southeast of Brazil. Physiology Journal. 2014:2014. 10.1155/2014/956960.

[30] Xiong C, Zhou A, Cao Z, Zhang Y, Qiu L, Yao C (2016). Association of pre-pregnancy body mass index, gestational weight gain with cesarean section in term deliveries of China. Sci Rep..

[31] Zachary Z, Brianna F, Brianna L, Garrett P, Jade W, Alyssa D (2020). Self-quarantine and weight gain related risk factors during the COVID-19 pandemic. Obes Res Clin Pract..

[32] Warren CS: How to curb emotional eating during the COVID-19 pandemic.https://www.psychologytoday.com/us/blog/naked-truth/202003/how-curb-emotional-eating-during-the-covid-19-pandemic. .

[33] Zhao A, zhang YM, Ke Y (2020). Emotional eating in pregnant women during the COVID-19 pandemic and its association with dietary intake and gestational weight gain. Nutrients.

[34] Hinman SK, Smith KB, Quillen DM, Smith MS (2015). Exercise in pregnancy: a clinical review. Sports Health..

[35] Stotland NE, Hopkins LM, Caughey AB (2004). Gestational weight gain, macrosomia, and risk of cesarean birth in nondiabetic nulliparas. Obstet Gynecol..

[36] Yu SWY, Hill C, Ricks ML, Bennet J, Oriol NE (2017). The scope and impact of mobile health clinics in the United States: a literature review. Int J Equity Health.

[37] Turrentine M, Ramirez M, Monga M, Gandhi M, Swaim L, Tyer-Viola L (2020). Rapid deployment of a drive-through prenatal care model in response to the coronavirus disease 2019 (COVID-19) pandemic. Obstet Gynecol..

