# Supplementary material for: The associated factors of cesarean section during COVID-19 pandemic: a cross-sectional study in nine cities of China
Source: Environ Health Prev Med. 2020 Oct 10;25:60. doi: 10.1186/s12199-020-00899-w (PMC7547292; doi:10.1186/s12199-020-00899-w)
Supplement: Supplementary file 1 — Additional file 1: Supplementary Table 1. Ways of women access to nutrition instructions among women living in the regions with different COVID-19 confirmed cases. Supplementary Table 2. Sensitivity analysis of influence factors of delivery mode.a [file 12199_2020_899_MOESM1_ESM.docx]

Supplementary Table 1 Ways of women access to nutrition instructions among women living in the regions with different COVID-19 confirmed cases

|  | Confirmed cases in living regions | | P |
| --- | --- | --- | --- |
|  | 500 or below | Over 500 |  |
| Nutrition clinic of hospital |  |  |  |
| No | 290(85.0) | 273(81.0) | 0.194 |
| Yes | 51(15.0) | 64(19.0) |  |
| Obstetrics clinic |  |  |  |
| No | 201(58.9) | 175(51.9) | 0.078 |
| Yes | 140(41.1) | 162(48.1) |  |
| Online instruction |  |  |  |
| No | 261(76.5) | 189(56.1) | <0.001 |
| Yes | 80(23.5) | 148(43.9) |  |
| Online courses |  |  |  |
| No | 299(87.7) | 249(73.9) | <0.001 |
| Yes | 42(12.3) | 88(26.1) |  |
| Combined ^a^ |  |  |  |
| No | 144(42.2) | 90(26.7) | <0.001 |
| Yes | 197(57.8) | 247(73.3) |  |

variables were presented as proportions and compared with Chi-square tests.

^a^ Women who got any instructions through the four approaches were regarded as getting nutrition instruction.

Supplementary Table 2 Sensitivity analysis of influence factors of delivery mode.^a^

| Variables | OR (95%CI) | P |
| --- | --- | --- |
| Age (years) |  |  |
| ≤30 | Ref |  |
| >30 | 1.70(1.20,2.40) | 0.003 |
| Pre-gestational BMI (kg/m^2^) | 1.15(1.09,1.22) | <0.001 |
| Number of COVID-19 cases in resident region |  |  |
| ≤500 | Ref |  |
| >500 | 2.57(1.83,3.65) | <0.001 |
| History of metabolic disease |  |  |
| No | Ref |  |
| Yes | 1.38(0.91,2.09) | 0.131 |
| Getting Nutrition instruction |  |  |
| No | Ref |  |
| Yes | 1.37(0.95,1.98) | 0.097 |
| Gestational weight gain |  |  |
| Optimal | Ref |  |
| Low | 1.31(0.85,2.03) | 0.221 |
| Excess | 1.74(1.18,2.58) | 0.005 |
| Education level |  |  |
| Middle school and below | Ref |  |
| College and above | 0.69(0.47,1.00) | 0.050 |
| getting regular prenatal check-ups |  |  |
| No | Ref |  |
| Yes | 1.27(0.91,1.79) | 0.166 |

Factors with P<0.1 in the univariate analysis were included in the multivariate logistic regression model.

BMI: body mass index; COVID-19: 2019-novel Coronavirus; OR: odds ratio; Ref: reference.

^a^ Education level (middle school and below or college and above) and getting regular antenatal care during the epidemic (no or yes) were involved in the multivariate logistic regression model.
